# Efficacy of Oral Versus Injectable Iron in Patients With Chronic Kidney Disease: A Two-Year Cross-Sectional Study Conducted at a Rural Teaching Hospital

**DOI:** 10.7759/cureus.27529

**Published:** 2022-07-31

**Authors:** Sachin Agrawal, Sharad Sonawane, Sunil Kumar, Sourya Acharya, Shilpa A Gaidhane, Anil Wanjari, Ruchita Kabra, Neha Phate, Abhinav Ahuja

**Affiliations:** 1 Internal Medicine, Jawaharlal Nehru Medical College, Datta Meghe Institute of Medical Sciences, Wardha, IND; 2 Epidemiology and Public Health, Jawaharlal Nehru Medical College, Datta Meghe Institute of Medical Sciences, Wardha, IND

**Keywords:** serum ferritin, transferrin saturation, hemoglobin, oral iron, intravenous iron, chronic kidney disease

## Abstract

Aim

Anaemia (particularly iron deficiency) is of important concern in patients with chronic kidney disease (CKD) as it reflects the outcome of the disease. Current recommendations for the use of intravenous iron (IV) therapy in the management of anaemia in such patients are limited. This study highlights the comparison of oral to intravenous iron in patients with chronic kidney disease.

Materials and methods

This is a prospective case-control study comparing intravenous iron to oral iron in chronic kidney disease patients admitted to the Medicine Department of Acharya Vinoba Bhave Rural Hospital, in central India from October 2018 to October 2020. A total of 150 patients were divided into two groups of 75 each, one receiving oral iron (ferrous sulfate 325 mg tablets) and the other intravenous iron (IV iron sucrose).

Results

Serum iron, serum ferritin, and transferrin saturation (TS) showed increased levels in the IV iron group than in the oral iron group. In the IV group, a statistically significant increase was found in haemoglobin levels after therapy among all stages of kidney disease (p<0.05) while the same was not reported in the oral iron group.

Conclusion

IV iron sucrose therapy had been found to be effective, well-tolerated, and more successful than oral iron treatment in chronic kidney disease patients as far as the parameter of iron deficiency anaemia is concerned.

## Introduction

With its high prevalence, morbidity, and mortality rate, chronic kidney disease (CKD) is a major public health concern [1]. Iron deficiency anaemia is found in all grades of chronic kidney disease but more in haemodialysis-dependent patients needing iron therapy. In haemodialysis-dependent chronic kidney disease, there are more favourable outcomes for injectable iron supplements over oral iron [2,3]. Clinical evidence had shown that the use of intravenous (IV) iron in patients with CKD is more effective than oral treatment, but in chronic kidney disease patients not on dialysis there is no widely accepted consensus on which route of iron administration should be used as first-line therapy. Moreover, the optimum route of giving iron to CKD patients is still controversial [3,4].

Key systems for the pathogenesis of pallor in chronic kidney disease include an overall lack of erythropoietin, iron inadequacy and malnutrition, expanded blood misfortune, and abbreviated erythrocyte life expectancy.

The successful treatment of CKD anaemia is accomplished with recombinant human erythropoietin. Several studies have shown that in almost all erythropoietin-treated patients, iron supplementation is needed because iron deficiency may contribute to erythropoietin hypo-responsiveness [4-8]. The utilization of intravenous (IV) iron can be restricted by anaphylactic responses due to dextran‑containing iron formulations [6,8].

Patients with CKD on haemodialysis are given iron therapy as an alternative to erythropoiesis-stimulating agents (ESAs); whereas only 33% of CKD patients not on haemodialysis receive ESA treatment [9]. Two multicenter trials in the United States sought to determine the efficacy and feasibility of iron sucrose infusion in the treatment of dialysis-related frailty. No true drug-related unfavourable effects or intense contact reactions were seen in either of these cases. Both these two trials were limited in scope and both had subtle differences in iron upkeep regimes. To date, no large-scale, multicenter, multi-portion analysis has been conducted to study the efficacy of any intravenous iron compound in a patient-administration environment expressed by various dosing regimens as rehashed dosages [8,9].

Due to the conflicting literature available, we conducted this study to analyse oral versus injectable iron therapy among CKD subjects not on erythropoietin and haemodialysis to know which is better.

## Materials and methods

This prospective cross-sectional study was conducted in the department of medicine at a rural teaching hospital in central India from October 2018 to October 2020. Patients above 16 years of age with a glomerular filtration rate of less than 60 ml/min/1.73m^2^ for more than three months duration, haemoglobin (Hb) <13.0 g/dL and <12.0g/dL in males and females respectively were included in the study. Patients were further categorized into mild anaemia (Hb 9.0-10.9 g/dL), moderate anaemia (Hb 7.0-8.9 g/dL), and severe anaemia (Hb less than 7.0 g/dL) according to the World Health Organization classification [7]. Patients with acute kidney injury, iron overload or iron use, drug aversion, past sensitivities, decompensated liver cirrhosis or active hepatitis (more than three times the upper average limit of alanine aminotransferase) and care by ESA within eight weeks before the screening visit were excluded from the study. The ethical committee approval letter number was DMIMS (DU)/IEC/2018-19/7552.

The sample size was calculated using the formula:

$n=(Z alpha/2)2 x P x (1-P)/d2$

where Z alpha/2: 1.96, P = prevalence of anaemia= 90.39%=0.9039, d = desired error of margin 4%=0.04.

According to the formula, 134 cases of chronic kidney disease was the sample size but we took 150 cases of chronic kidney disease, distributed equally into two groups of 75 cases, one where the participants received infusions of IV iron sucrose and another group where the participants received oral ferrous sulfate (325 mg tablets). Eligible participants were randomized in a 1:1 ratio, the sequence of which was computer-generated by a statistician.

Besides the complete blood count, their transferrin saturation (TS), serum ferritin, total iron binding capacity (TIBC) and Hb levels were measured. Patient with overnight fasting and who was not on iron supplements for a minimum of seven days were preferred prior to sample collection.

Dose

Patients were instructed to take ferrous sulfate 325 mg tablets (65 mg elemental iron) with water orally one hour before meals three times a day for 30 days. IV iron sucrose 200mg diluted with 200 ml of 0.9% sodium chloride, infused over 30-60 min. IV iron sucrose 200 mg dose given once a week for four weeks.

Statistical analysis

Data collected were tabulated in a Microsoft Excel sheet. The means and standard deviations of the measurements per group were used for statistical analysis (SPSS v. 22.00 for Windows; IBM Corp., Armonk, NY). The difference between the two groups was performed by t-test with the p-value set at < 0.05.

## Results

Males (58.7%, 66.7%) were present in greater numbers than females (41.3%, 33.3%) in both groups. The majority of subjects belonged to 41-60 years of age in the IV iron group (33) as well as in the oral iron group (36). Mild, moderate and severe Hb was reported among 24%, 56%, 20% and 20%, 58.67%, and 21.33% of the subjects in the IV and oral iron group respectively as shown in Table 1.

**Table 1:** Baseline characteristics among the study subjects SD: standard deviation; Hb: haemoglobin

Variables	IV Iron Group (N=75)	Oral Iron Group (N=75)
Male	44 (58.7%)	50 (66.7%)
Female	31 (41.3%)	25 (33.3%)
Age Group (in years)		
20-30	9 (12%)	10 (13.33%)
31-40	18 (24%)	11 (14.67%)
41-50	15 (20%)	20 (26.67%)
51-60	18 (24%)	16 (21.33%)
>60	15 (20%)	18 (24%)
Age (in years) ± SD	47.55 ± 13.94	49.40 ±14.79
Weight (in Kg) ± SD	65.35 ±12.29	62.81 ±12.86
Severity of anaemia (Hb levels)		
Mild	18 (24%)	15 (20%)
Moderate	42 (56%)	44 (58.67%)
Severe	15 (20%)	16 (21.33%)

After IV and oral iron infusion, Hb levels increased in all the stages of anaemia. In moderate anaemia cases, before and after therapy, Hb levels increased from 7.21 to 10.04 gm/dl in the IV group (p<0.05) while in the oral iron group, it increased from 7.54 to 8.33 (p>0.05). Before and after therapy in the IV group, Hb increased from 5.12 to 7.71 and 5.37 to 6.35 in the oral group among severe anaemia cases. When Hb levels were compared statistically before and after therapy in severe anaemia cases in the iron as well as oral group, it was found to be statistically significant as shown in Table 2.

**Table 2:** Mean haemoglobin in both groups after oral and IV infusions according to anaemia grades *statistically significant

Grades	IV Iron Group (N=75)		Oral Iron Group (N=75)	
	Mean±SD		Mean±SD	
	Before therapy	After therapy	Before therapy	After therapy
Mild anaemia	10.46±0.65	12.18±0.99	10.35±0.88	11.00±1.17
p-value	0.02*		0.09	
Moderate anaemia	7.21±1.00	10.04±1.38	7.54±0.84	8.33±0.80
p-value	<0.01*		0.16	
Severe anaemia	5.12±0.61	7.71±1.14	5.37±0.40	6.35±0.56
p-value	0.008*		0.04*	

All the parameters viz. serum iron, serum ferritin, and TS increased comparatively more in IV iron as compared to the oral iron group. Before therapy, the mean TIBC was 395.65 and 399.55 in the IV and oral iron groups respectively. After therapy, mean TIBC decreased to 345.11 and 371.04 in the IV and oral iron groups respectively. When the TIBC mean was compared statistically in IV iron and oral groups, it was found to be statistically significant as shown in Table 3.

**Table 3:** Serum iron, serum ferritin, and transferrin saturation comparison before and after infusion among the groups * statistically significant TIBC: total iron binding capacity

Variables	IV Iron group		Oral Iron group	
	Mean±SD		Mean±SD	
	Before therapy	After therapy	Before therapy	After therapy
Serum iron	91.60±29.94	132.47±48.75	88.39±28.92	127.97±52.39
p-value	0.004*		0.009*	
Serum ferritin	112.23±43.18	139.29±51.72	122.49±57.28	133.72±56.79
p-value	0.002*		0.002*	
Transferrin saturation	18.11±3.12	25.33±3.58	17.80±3.17	21.61±3.46
p-value	<0.01*		0.02*	
TIBC	395.65±35.72	345.11±31.26	399.55±23.37	371.04±23.37
p-value	0.003*		0.02*	

Table 4 shows the Hb comparison according to CKD stage pre and post-infusion among the groups. In the IV Iron group, a statistically significant increase was found in Hb after the therapy among all stages of kidney disease (p<0.05) while the same was not reported in the oral iron group.

**Table 4:** Haemoglobin comparison according to CKD stage pre- and post-infusion among the groups *: statistically significant

Stages	IV Iron		Oral Iron	
	Before Therapy	After Therapy	Before Therapy	After Therapy
Stage 3a	8.5±1.97	11.5±2.08	8.33±2.41	9.16±2.65
p-value	<0.01*		0.09	
Stage 3b	7.28±2.05	9.52±1.58	7.63±1.39	8.24±1.39
p-value	0.02*		0.22	
Stage 4	7.19±1.98	10.10±1.57	7.77±1.93	8.69±1.75
p-value	<0.01*		0.07	
Stage 5	6.38±1.18	8.54±1.25	7.55±1.75	8.31±1.55
p-value	0.02*		0.16	

Gastrointestinal side effects were reported more among the oral iron group in comparison to the IV iron group while side effects like headache, myalgia, hypotension and infusion site reactions were found more in the IV group as shown in Table 5.

**Table 5:** Comparison of side effects among both the groups.

Complications	IV Iron	Oral Iron
	N (%)	N (%)
Gastrointestinal disorders		
Constipation	13 (17.3%)	35 (46.7%)
Diarrhoea	3 (4.0%)	5 (6.7%)
Discoloured faeces	0	5 (6.7%)
Gastrointestinal haemorrhages	0	3 (4%)
Others		
Nausea	5 (6.7%)	6 (8%)
Headache	4 (5.3%)	3 (4%)
Myalgia	5 (6.7%)	0
Hypotension	3 (4.0%)	0
Infusion site reactions	5 (6.7%)	0

## Discussion

Appropriate treatment of CKD anaemia can contribute to considerable improvement in cardiovascular status and decreased hospitalization rate [10]. Iron-deficient anaemic CKD patients have been recommended to take oral or IV iron supplements according to current National Kidney Foundation Kidney Disease Outcome Quality Initiative guidelines. Some studies have already demonstrated that the efficacy of oral iron is as good as that of IV iron [11,12]. However, promising results were shown in terms of IV iron, not only increasing the Hb level but also replenishing the iron stores [13,14]. We have observed that IV iron is much more efficacious in raising the Hb level as the primary endpoint compared to oral iron at the end of four weeks. There are reports on the treatment of anaemia with IV iron, but there is little comparable oral therapy evidence, as well as contradictory data [14,15].

In our study, after IV and oral iron infusion, Hb levels increased in all the stages of anaemia. In moderate anaemia cases, before and after four weeks of therapy, Hb levels increased from 7.21 to 10.04 gm/dl in the IV group (p<0.05) while in the oral iron group, it increased from 7.54 to 8.33 (p>0.05). Before and after therapy in the IV group, Hb increased from 5.12 to 7.71 and 5.37 to 6.35 in the oral iron group among severe anaemia cases with a statistically significant difference. When overall mean Hb was compared, it was found to be statistically significant in the IV group.

Charytan et al. observed important mean changes in baseline Hb values for each visit in all treatment groups in their study, with a mean change from baseline to day 43 of 1.0 g/dl (p=0.0001) for IV iron and 0.7 g/dl (p=0.0001) for oral iron, respectively. The mean change in Hb values from baseline to day 43 was higher for IV iron, but this discrepancy did not achieve statistical significance. In the oral group, the mean maximum Hb level was 10.7 g/dl, and in the IV group, 11.1 g/dl [16].

In a study by Adhikary et al. mean Hb increment was more in the IV iron group than in the oral iron group. Around 60% of patients in the IV iron group had an increase in the Hb level of more than 1gm/dl while only 20% of the oral iron group had this increase [17].

In our study, all the parameters like serum iron, serum ferritin, and TS increased comparatively more in IV group as compared to the oral iron group. These results were in accordance with the study done by Charytan et al. They showed that the IV iron array had a critical mean rise in ferritin estimates with a mean shift of 288.0 ng/ml (p=0.0001) from normal to day 43 [16].

Agarwal et al. analyzed seventy-five patients (intravenous iron n = 36, oral iron n = 39) and showed that the change from baseline in Hb was similar in the two groups; however, the increase in Hb was significant with intravenous iron. In comparison to oral iron, intravenous iron achieved greater improvements in ferritin [18].

In a meta-analysis of six studies by Rozen-Zvi et al., five trials revealed a mean increase in Hb with intravenous iron was 0.31 (95% CI 0.09 to 0.53) g/dL; while one had a mean decrease in Hb of 0.52 g/dL associated with intravenous iron administration as compared to oral iron [19].

IV iron was more effective than oral with respect to the elevation of Hb, ferritin and TSAT levels in patients with CKD on dialysis than those not on dialysis. Patients who were on IV iron had a more rapid response in achieving target Hb levels [20-23].

In this study gastrointestinal disorders were reported more in the oral iron group (n=48) as compared to the IV iron group (n=16) while side effects like headache, myalgia, hypotension and infusion site reactions were found more in the IV group. These results were in accordance with other studies [7,8,24,25].

Why are more studies required?

Two famous studies (FIND-CKD and REVOKE study) having significant advances over previous randomized trials in terms of larger numbers of patients and much longer follow-up periods, revealed contrasting conclusions. REVOKE trial concluded that oral iron may be the preferred first-line treatment for iron deficiency anaemia in CKD patients, while the FIND-CKD study revealed that the use of IV iron to target higher ferritin levels may contribute to improved anaemia management in this patient population [7,8].

Various other studies had compared oral to intravenous iron for managing anaemia in patients with chronic kidney disease who were not on dialysis and concluded with increases in both Hb and ferritin following IV iron therapy rather than with oral iron [21-30].

The intravenous route of iron administration may be a preferred route, more so in the Indian context where the prevalence of iron deficiency anaemia in chronic kidney disease is fairly high [23].

The main limitation of this study was the small sample size, short-term follow-up period as well as non-inclusion of haemodialysis patients.

## Conclusions

The use of iron in the management of anaemia associated with CKD has been recommended keeping into account the route of administration, and selection of treatment regimen. Along with this, a number of factors like severity of anaemia, treatment goals, CKD stage and dialysis modality must be considered as practical guidelines. Though there is limited evidence on the use of IV versus oral iron in patients with CKD, as per the KDIGO guideline, findings from a number of studies assessing the efficacy of IV iron have been published, including this article. IV iron may be the preferred initial treatment option for physicians wanting to increase Hb concentrations or delay alternative anaemia management in patients with chronic kidney disease.
